# Comparison of Signal and Gap-Detection Thresholds for Focused and Broad Cochlear Implant Electrode Configurations

**DOI:** 10.1007/s10162-015-0507-y

**Published:** 2015-02-03

**Authors:** Julie Arenberg Bierer, John M. Deeks, Alexander J. Billig, Robert P. Carlyon

**Affiliations:** 1 Department of Speech and Hearing Sciences, University of Washington, 1417 NE 42nd St., Seattle, WA 98105 USA; 2 Cognition and Brain Sciences Unit, Medical Research Council, 15 Chaucer Road, Cambridge, CB2 7EF UK

**Keywords:** cochlear implants, gap detection, psychophysics, electrode configuration

## Abstract

Cochlear implant (CI) users usually exhibit marked across-electrode differences in detection thresholds with “focused” modes of stimulation, such as partial-tripolar (pTP) mode. This may reflect differences either in local neural survival or in the distance of the electrodes from the modiolus. To shed light on these two explanations, we compared stimulus-detection thresholds and gap-detection thresholds (GDTs) at comfortably loud levels for at least four electrodes in each of ten Advanced Bionics CI users, using 1031-pps pulse trains. The electrodes selected for each user had a wide range of stimulus-detection thresholds in pTP mode. We also measured across-electrode variations in both stimulus-detection and gap-detection tasks in monopolar (MP) mode. Both stimulus-detection and gap-detection thresholds correlated across modes. However, there was no significant correlation between stimulus-detection and gap-detection thresholds in either mode. Hence, gap-detection thresholds likely tap a source of across-electrode variation additional to, or different from, that revealed by stimulus-detection thresholds. Stimulus-detection thresholds were significantly lower for apical than for basal electrodes in both modes; this was only true for gap detection in pTP mode. Finally, although the across-electrode standard deviation in stimulus-detection thresholds was greater in pTP than in MP mode, the reliability of these differences—assessed by dividing the across-electrode standard deviation by the standard deviation across adaptive runs for each electrode—was similar for the two modes; this metric was also similar across modes for gap detection. Hence across-electrode differences can be revealed using clinically available MP stimulation, with a reliability comparable to that observed with focused stimulation.

## **INTRODUCTION**

Cochlear implants (CIs) have successfully restored hearing to more than 300,000 patients worldwide. Although many listeners achieve excellent open-set speech perception, even in the absence of visual information, performance varies widely across listeners. The primary source of this variability remains a matter of debate, but one hypothesis is that the within-listener variability in perception for stimulation from individual CI channels reflects an underlying pathology that impairs speech perception.

Psychophysical measures obtained using focused electrical stimulation vary substantially across CI listeners and across electrodes within listeners. Previous studies have shown that channels with detection thresholds higher than the average for that listener exhibit poorer spectral/spatial resolution (Bierer and Faulkner 2010; Long et al. 2014) and smaller dynamic ranges (Bierer and Nye 2014). Further, high variability in threshold from channel to channel within individuals measured with monopolar (MP) (Pfingst and Xu 2004) and/or with focused stimulation (Pfingst and Xu 2004; Bierer 2007; Long et al. 2014) is associated with poor speech perception scores. Those findings suggest that speech perception may be degraded by the poor transmission of information by a subset of electrodes and that detection thresholds may provide some indication of the presence and identity of those channels. However, it is possible that more than one factor influences the detection threshold for stimulation of a given electrode and that these factors differ in the extent to which they influence the transmission of speech information. Specifically, detection thresholds may be affected by both degeneration of neurons close to the stimulating electrode and/or by the distance of that electrode from the modiolus (Bierer 2007; Long et al. 2014). Although both of these factors would lead to broader excitation patterns, it is possible that this could be alleviated by the use of more focused stimulation in the case of large modiolar distance, but not when local neural survival is poor.

In everyday use, loudness is, to a first approximation, equated across electrodes in a patient’s clinical map by the assignment of different thresholds (T) and most comfortable levels (MCLs). However, substantial across-electrode differences in suprathreshold tasks may remain (Kong et al. 2009; Pfingst 2011; Garadat et al. 2012). The present study investigates the across-electrode variation in temporal information conveyed by CI electrodes. We measured gap-detection thresholds (GDTs), which we believe represent a relatively pure measure of temporal sensitivity. GDTs have been modeled using the concept of a sliding temporal window followed by the detection of changes in the level of the output of that window (Plack and Moore 1990). In normal hearing, the equivalent rectangular duration of that window is less than 10 ms, and a similar value has been used to model data from CI users, albeit with a different experimental paradigm (McKay and McDermott 1998). Detection of such short gaps will be affected by both differences in the duration of the temporal window and/or by differences in the fidelity of the auditory nerve response that, presumably, forms the input of that window. Another task that has been proposed as a measure of temporal sensitivity is the detection of amplitude modulation (modulation detection thresholds (MDTs)). Those tasks typically use low modulation rates, for example, 10 Hz, for which the corresponding period is much longer than 10 ms (Garadat et al. 2012). We believe that those tasks may be more sensitive to the encoding of amplitude differences than to differences in the duration of the temporal window, and, as the modulation period gets longer, the task becomes more and more like one of level discrimination. Yet another task, rate discrimination, does involve the processing of short temporal intervals, but may additionally involve pitch processing.

We hypothesize that gap detection might be impaired in some channels by the altered temporal discharge patterns associated with neural degeneration. Shepherd and Javel (1997) reported that the deafened auditory nerve could produce a “bursting” response to high-rate electrical stimulation and showed that this occurred for cats that had been deaf for 2 months, but not in a cat that was deafened immediately prior to testing. If elevated detection thresholds, particularly in focused stimulation mode, are largely indicative of poor neural survival, and if the effects of that poor survival are similar for stimulus detection and gap detection, then we would expect performance on the two tasks to correlate across electrodes. On the other hand, it is possible that no such correlation will be found, as has previously been reported for MDTs (Pfingst 2011). This could occur if elevated detection thresholds were primarily due to larger electrode-modiolar distances rather than poor local neuron survival. Alternatively, no correlation would be observed if, for example, some electrodes excited regions where AN fibers were sparse but responded with good temporal fidelity, whereas others excited a region where fibers were more numerous but that produced a temporally degraded response.

We measured both detection thresholds and gap-detection thresholds (GDTs) using MP and a focused partial-tripolar (pTP) mode of stimulation, across a subset of electrodes in ten implanted ears from nine CI users. Our results show that both measures could vary substantially across electrodes within a given subject and that, for a given measure, there was a highly significant correlation between the two modes of stimulation. However, there was no correlation between the two tasks, suggesting that GDTs are influenced by factors separate from, or additional to, those reflected in detection thresholds.

A second goal was to evaluate across-electrode variation in MP mode. Although large across-electrode variations in detection threshold have been obtained with focused stimulation, smaller variations are typically obtained in MP mode (Pfingst and Xu 2004; Bierer 2007; Long et al. 2014). However, substantial across-electrode differences have been observed in MP mode with suprathreshold tasks such as rate discrimination (Kong et al. 2009) and modulation detection (Pfingst 2011; Garadat et al. 2012). Indeed, Garadat and Pfingst (2011) have reported across-electrode variations in MP GDTs, when each electrode was stimulated at the same percentage of its dynamic range, although differences were greatly reduced when electrodes were compared at the same loudness. To minimize such loudness effects, we measured GDTs for stimuli presented at the same loudness, namely the MCL. We compared the amount of across-electrode variation in MP and focused (pTP) stimulation. To do so, we computed a metric that controlled for any possible differences in the absolute size of the GDTs between the two modes. The metric was defined as the ratio between two values: the *between*-*electrode* standard deviation, calculated from the standard deviation of the mean GDTs from the four adaptive runs used for each electrode, and the *within*-*electrode* standard deviation, obtained by calculating the standard deviation across adaptive runs for each electrode separately and then averaging these standard deviations across electrodes. This dimensionless metric, termed the standard deviation ratio (“SDR”), could also be modified to describe the across-electrode variation in stimulus-detection thresholds, or to compare the across-electrode variation between different tasks, such as detecting a pulse train and detecting a gap. In addition, it provides an arguably more valid method than the simple across-electrode standard deviation, when comparing the across-electrode variation in detection thresholds between the two modes. This is because the same across-electrode standard deviation may not correspond to the same amount of variation in sensitivity in the two modes, if the slopes of the underlying psychometric functions are different. A difference in slope should, however, affect both the within- and between-electrode standard deviation, and so the ratio between these two values may be more appropriate than the across-electrode standard deviation alone.

## **METHODS**

### Subjects

Nine postlingually deafened adults wearing the Advanced Bionics HiRes 90K CI participated; their details are shown in Table 1. One subject, who was bilaterally implanted, was tested in each ear and is listed as S30L and S39R in the table. This subject’s two ears were treated as completely separate, and therefore, for the purposes of analysis and for discussion in the remainder of this article, there were ten “subjects.” Five of the subjects were implanted and tested in Cambridge, England, UK, whereas the other half was implanted and tested in Seattle, WA, USA. All procedures were approved by the respective Human Subjects Review Boards.

**TABLE 1:** Details of the subjects who took part in the experiments. Subject codes starting with the letter “S” refer to subjects implanted and tested in Seattle (WA, USA), whereas those starting with “C” were implanted and tested in Cambridge, England.

Subject	Age (years)	Onset age of severe hearing loss	Duration of CI use	Etiology
S22	72	55	4 years 9 months	Hereditary
S27	83	55–60	4 years 7 months	Unknown
S28	74	26	4 years 5 months	Hereditary
S30L	49	16	9 years	Hereditary
S39R	49	16	19 years (1) 8 years (2) 2 years (3)	Hereditary
C1	67	32	3 years	Unknown
C2	31	7	2 years	Unknown
C3	69	50	2 years	Otosclerosis
C4	66	37	4 years	Otosclerosis
C5	53	Prog since 1992	4 years	Unknown

Note that S39R has been implanted in that ear three times as a result of device failures

### Stimuli

Biphasic, charge-balanced, cathodic-phase-first pulses were used. Phase durations were either 97 or 194 μs, and the pulse rate was 1031 pps. The longer phase duration was used when MCL could not be reached within the compliance limits for the shorter phase duration. MCLs were obtained with individual pulse train presentations. Following each presentation, the subject indicated the loudness of the train using the Advanced Bionics loudness rating scale for which a “6” is “most comfortable” and a “7” is “loud but comfortable.” The level was increased in 0.5 or 0.1 dB steps until the rating was “7” and then reduced until the listener reported a “6” again. MCLs were not loudness balanced. Pulse train durations were 200 ms for signal detection and 400 ms for gap detection. Stimuli were either presented in the MP or the pTP configuration with a return current fraction (*σ*) of 0.75. All stimuli were presented and controlled using research hardware and software (“BEDCS”) provided by the Advanced Bionics company. Programs were written using the MATLAB programming environment, which controlled low-level BEDCS routines. Stimuli were checked using a test implant and digital storage oscilloscope. The same software and identical hardware were used in both testing sites (Cambridge and Seattle).

Signal detection thresholds were measured using a three-down, one-up, two-interval forced-choice adaptive procedure that converged on 79 % correct. Step size was 1 dB for the first two turnpoints and 0.25 dB thereafter. The mean of the last four of six reversals was used to estimate threshold. Four or five repetitions were performed for each measurement. Thresholds were measured in pTP mode for all available channels (usually 2 through 15). Subjects were asked “Which interval contained the sound?” and responded using a computer mouse. Note that subject C3 was not tested on electrodes 7, 8, and 9 because those channels were deactivated from that subject’s everyday program. The same is true for subject C5 for electrode 15. Because of time constraints and health issues with subject S27, only a subset of electrodes was tested. We had previously tested all 14 electrodes for S27 but with a different pTP fraction of 0.9, so the highest and lowest threshold channels from these earlier measures were purposely included.

For each subject, four or five electrodes were selected for further testing, such that at least two had low pTP thresholds and at least two had high pTP thresholds. The general rule was to select the two highest and two lowest thresholds, unless this involved two adjacent “high” or “low” electrodes. Detection thresholds for these additional electrodes were obtained in MP mode, using the same method as described above for pTP stimulation. GDTs were obtained in both modes using an adaptive procedure similar to that used for the threshold measurements. Each run started with an easily discriminable gap size that was then adjusted in steps of 40 and 10 % of the gap durations for the first two and last four turnpoints, respectively. The threshold for each run was determined from the arithmetic mean of the last four turnpoints. Four or five runs were performed for each measurement, and the threshold for that measurement was calculated from the arithmetic mean of those runs. Subjects were asked to answer, “Which interval contained the gapped sound?” For the gap-detection task, the pulse train duration was roved by +/− 10 %, in order to reduce the usefulness of duration as a cue. The stimulus level was not roved. For both the GDT and detection threshold measures, correct-answer feedback was provided after every trial.

### Loudness Balancing

Gap stimuli were presented at each individual’s MCL with both the MP and pTP electrode configurations, when possible. In one case, the most comfortable level could not be reached because of the compliance limits of the system, and so a “soft” level was used in both modes and loudness balancing of stimuli across channels was performed. For this one subject (C2), the procedure was first to determine the highest level reachable within the compliance limits and comfort levels for all test electrodes. The lowest of those current levels was then used for loudness balancing. The subject set the level of all test electrodes to be at the same subjective level on the loudness rating scale. Then one of the low-threshold electrodes was selected to be the reference, and all of the other electrodes were adjusted to match the perceived loudness of the reference. The subject had manual control over the level of the second sound with either 0.25 (“+” or “−”), 0.5 (“++” or “− −”), or 1 dB (“+++” or “− − −”) step sizes. When the listener believed the two sounds to be equally loud, they clicked on a “Done” button. For half the matches, the starting level was well below the loudness-matched level predicted from loudness ratings; for the other half, the starting level was above this estimated value. Following four repetitions of loudness balancing for each test electrode, the reference electrode became the test electrode. The procedure was repeated four times for each reference electrode.

## **RESULTS**

### Signal Detection Thresholds

**FIG. 1:**
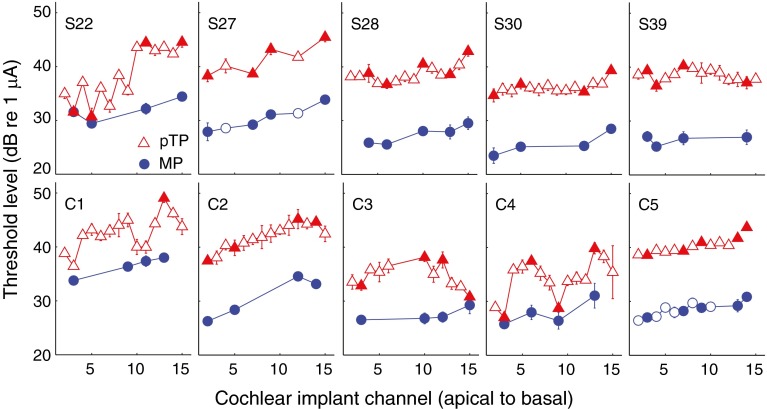
Single-channel behavioral thresholds measured using the MP (*circles*) and partial-tripolar (*triangles*) configurations for all subjects. Each *panel* plots the single-channel detection thresholds for a given subject (indicated in the *top left corner of each panel*). The *abscissa* represents CI channel from apical to basal, and the *ordinate* represents detection threshold in decibels relative to 1 μA. The *filled symbols* indicate those channels selected for gap-detection testing. *Error bars* represent the standard deviation and are mostly smaller than the symbol size.

Figure 1 shows the mean detection thresholds for each subject and channel with the pTP and MP modes. Although thresholds are displayed for multiple electrodes in pTP mode, all analyses described throughout this paper are restricted to the four or five “main” electrodes, shown by the filled symbols, for each subject. Thresholds were lower for more apical stimulation in both stimulation modes. This was evaluated, for each mode separately, by entering the threshold data into a univariate Analysis of Covariance (ANCOVA), with subject as a fixed factor and electrode number as a covariate. The resulting correlation coefficients were 0.62 and 0.83 for the pTP and MP modes, respectively (*p* < 0.001, *df* = 31 in each case). Lower thresholds for apical stimulation have previously been reported for focused stimulation by Bierer (2007). Throughout this article, we calculate correlation coefficients from the ANCOVA from √(SUMSQ_covar_/(SUMSQ_covar_ + SUMSQ_error_)), where SUMSQ_covar_ and SUMSQ_error_ are the sums of squares for the covariate and error terms, respectively (Bland and Altman 1995).

**FIG. 2:**
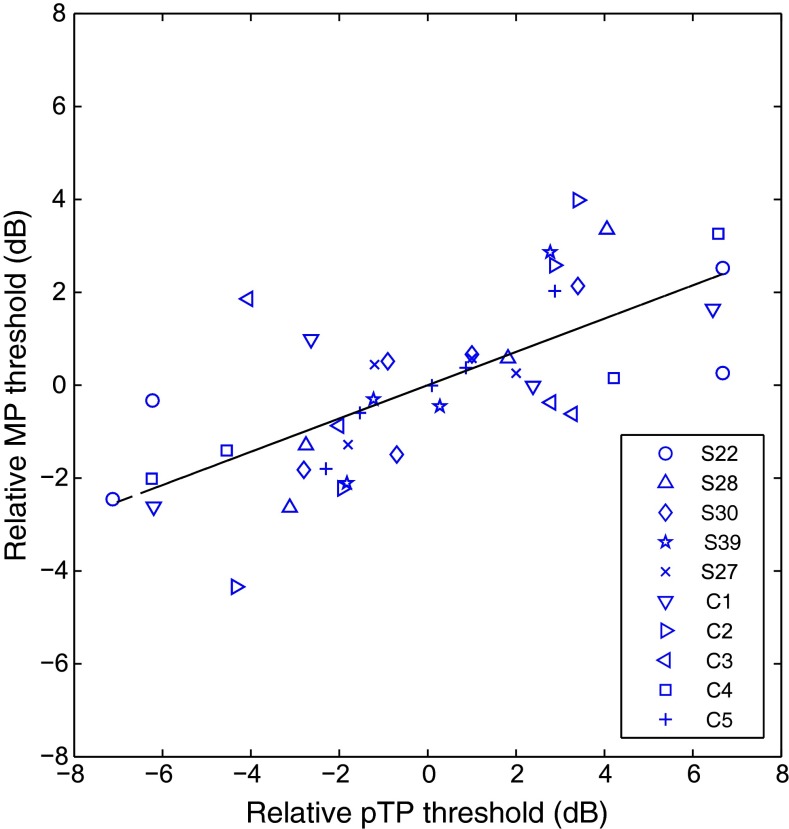
Relative MP thresholds (*ordinate*) as a function of partial-tripolar thresholds (*abscissa*). Thresholds are plotted relative to the within-configuration, average threshold for each subject and plotted in units of decibels. Subject is indicated by *symbol*. The *black line* depicts the least-squared error fit to the data.

Comparisons of pTP and MP signal detection thresholds are shown in Figure 2. Our analyses always partialed out the between-subject source of variation in the two measures, so as to reveal only the covariation across electrodes within each subject. Figure 2 therefore shows normalized thresholds for each mode, calculated by subtracting from each measure the average threshold across all electrodes for that subject and mode of stimulation. The relative MP thresholds (ordinate) are plotted as a function of relative pTP thresholds (abscissa). The correlation apparent in these normalized data was quantified by analyzing the un-normalized data using a univariate ANCOVA with MP threshold as the dependent variable, listeners as a between-subject factor, and pTP threshold as a covariate. This revealed a highly significant correlation (*r*(31) = 0.66, *p* = 0.001). Stimulus levels for GDT experiments are shown in Table 2. Note that, as shown in Table 3, the standard deviation across electrodes was substantially larger in pTP mode (3.6 dB) than in MP mode (1.9 dB; two-tailed *t* test, *df* = 8, *p* < 0.02). The correlation therefore suggests that the variation apparent in pTP mode is reflected in attenuated form in the MP thresholds. Interestingly, because the within-electrode standard deviation varied somewhat across subjects and modes of stimulation, the SDR did not differ significantly between the two modes (Table 3). Hence, we have no evidence that pTP thresholds produce a more *reliable* variation across electrodes. This is perhaps even more surprising given that the electrodes were selected for each subject on the basis of having markedly different pTP thresholds.

**TABLE 2:** Presentation levels for GDT experiments for each subject and electrodes, in dB re 1 μA, for pTP and MP modes

Subject	Electrode number (pTP; MP presentation level)				
S22	3 (50.1; 41.8)	5 (54.4; 43.9)	11 (55.5; 44.4)	15 (54.1; 44.2)	NA
S27	2 (55.0; 41.1)	7 (53.7; 41.9)	9 (57.4; 42.2)	15 (54.7; 43.7)	NA
S28	4 (48.0; 36.6)	6 (46.0; 36.7)	10 (47.1; 37.6)	13 (45.8; 37.9)	15 (45.8; 38.1)
S30	2 (46.2; 44.2)	5 (47.3; 43.5)	12 (49.6; 44.6)	15 (46.3; 43.1)	NA
S39	3 (53.9; 41.7)	4 (52.3; 41.7)	7 (53.7; 42.9)	14 (51.4; 42.5)	NA
C1	3 (52.9; 42.3)	9 (55.2; 44.3)	11 (54.1; 44.6)	13 (55; 44.6)	NA
C2	2 (45.5; 34.5)	5 (48.1; 37.0)	12 (53.1; 41.3)	14 (52.6; 41.2)	NA
C3	3 (48.3; 38.4)	10 (50.1; 41.6)	12 (48.9; 40.0)	15 (43.5; 38.1)	NA
C4	3 (46.2; 38.1)	6 (52.6; 41.0)	9 (49.5; 39.6)	13 (52.1; 40.4)	NA
C5	3 (50.4; 39.6)	7 (50.9; 40.0)	9 (51.2; 40.4)	13 (48.8; 39.1)	14 (50.0; 39.6)

**TABLE 3:** Across- and within-electrode standard deviations, and the ratios between them, for stimulus-detection thresholds

	Partial tripolar (*σ* = 0.75) detection threshold (dB)			Monopolar (*σ* = 0) detection threshold (dB)		
Subject	Within-channel	Across-channel	Std Dev	Within-channel	Across-channel	Std Dev
	Std Dev	Std Dev	Ratio	Std Dev	Std Dev	Ratio
S22	1.00	7.34	7.32	0.40	2.49	6.15
S27	0.87	3.51	4.06	0.68	2.64	3.89
S28	0.93	2.36	2.54	0.80	1.25	1.56
S30	0.78	2.06	2.65	0.58	2.11	3.64
S39	0.74	1.81	2.43	0.78	0.85	1.08
C1	1.28	5.61	4.39	0.72	1.87	2.59
C2	1.09	3.73	3.43	0.54	3.92	7.28
C3	1.05	2.78	2.63	0.97	1.26	1.29
C4	0.45	4.81	10.63	0.87	1.05	1.20
C5	0.69	1.99	2.88	0.88	1.61	1.83
Average	0.89	3.60	4.30	0.72	1.90	3.05

### Gap-Detection Thresholds

**FIG. 3:**
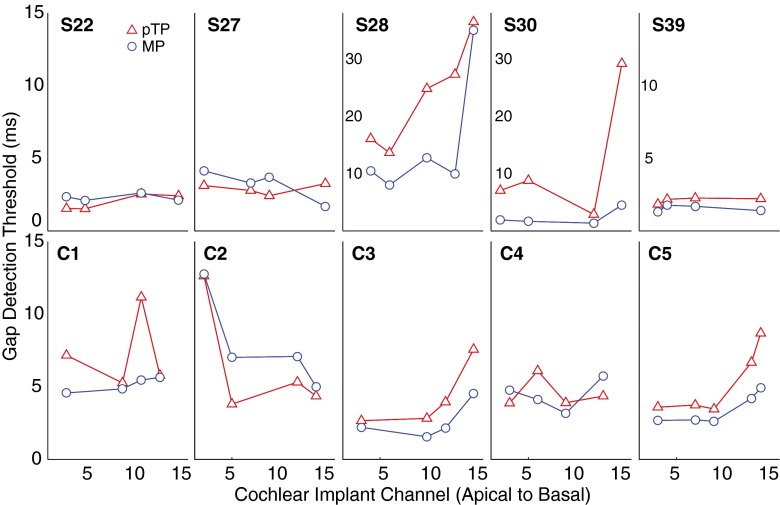
Single-channel GDTs measured using the MP (*circles*) and partial-tripolar (*triangles*) configurations for all subjects. The *abscissa* represents CI channel from apical to basal, and the *ordinate* represents GDTs in milliseconds. Note that for subjects S28 and S30, the ordinate scale is larger as denoted inside the *panels*.

**FIG. 4:**
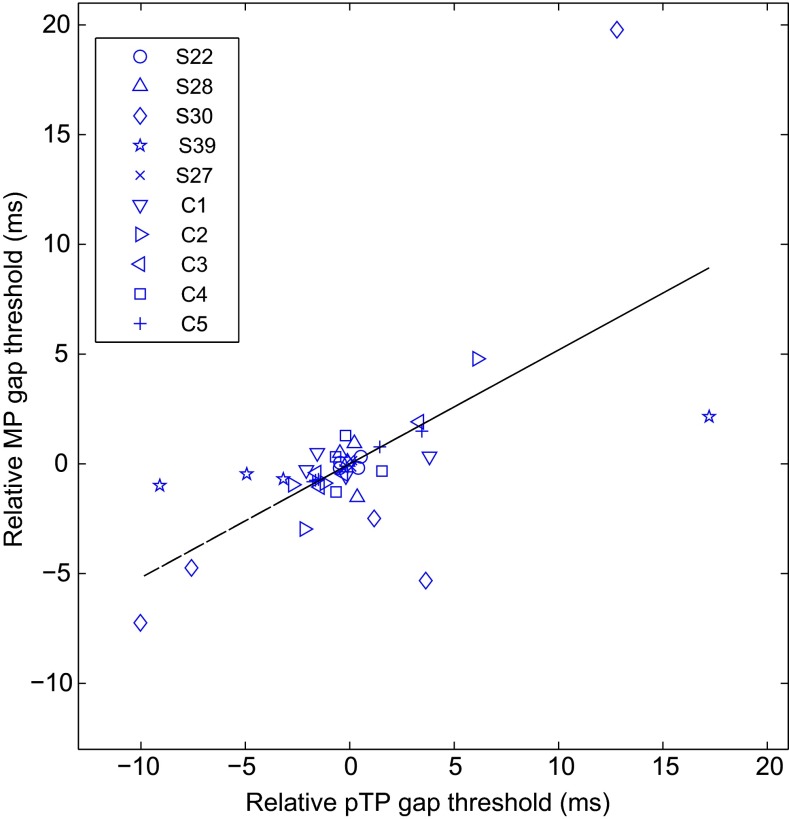
Relative MP GDTs (*ordinate*) as a function of partial-tripolar GDTs (*abscissa*). Gap thresholds are plotted relative to the within-configuration, average GDT for each subject and plotted in units of decibels. Subject is indicated by *symbol*. The *black line* depicts the least-squared error fit to the data.

GDTs for each subject are shown as a function of CI channel in Figure 3. For some subjects, GDTs are similar for the four or five tested electrodes, while for other subjects, a large range of GDTs was observed. Univariate ANCOVAs with subjects as fixed factor, GDT as dependent variable, and electrode as covariate revealed a significant tendency for GDTs to be lower at the apex in pTP mode (*r*(31) = 0.46, *p* = 0.03); the corresponding analysis for MP mode was not significant (*r*(31) = 0.19, *p* = 0.28). As for the threshold data, there was a strong relationship between GDTs in the two modes; a univariate ANCOVA with MP GDT as the dependent variable, subjects as fixed factor, and pTP GDT as the covariate revealed a highly significant correlation (*r*(31) = 0.65, *p* < 0.001). The correlation is also illustrated in Figure 4, which shows the relative GDTs obtained with pTP (abscissa) and MP (ordinate) configurations. Finally, we note that GDTs were significantly longer in pTP (mean = 7.7 ms) than in MP mode (5.1 ms), as revealed by an ANOVA with mode as a between-subject factor and subject as a within-subject factor. There were highly significant main effects of mode (*F*(1,32) = 12.71; *p* = 0.001) and subject (*F*(9,32) = 6.89; *p* < 0.001). The effect of mode differed across subjects, as indicated by a significant interaction (*F*(9,32) = 3.69; *p* < 0.005).

### Comparison of Signal and Gap-Detection Thresholds

**FIG. 5:**
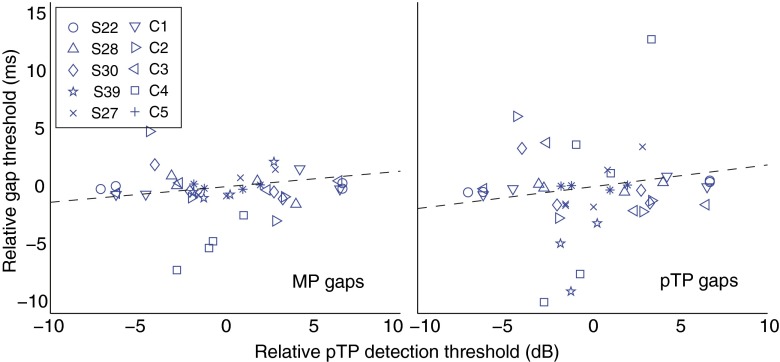
On the ordinate, relative GDTs for MP (*left panel*) and pTP (*right panel*) configurations are plotted as a function of partial-tripolar detection thresholds (*abscissa*). Subject is indicated by *symbol*.

Figure 5 plots the relationship between the normalized thresholds in pTP mode and the normalized GDTs in both the pTP and MP modes. Neither part of the figure provides strong evidence for a significant correlation. The absence of a relationship between the two detection measures was confirmed by univariate ANCOVAs, with GDT as dependent variable, subjects as fixed factor, and threshold as covariate. These analyses were carried out separately for each mode and revealed nonsignificant correlations between the normalized pTP detection threshold and the MP (*r*(31) = 0.13, *p* = 0.46) and pTP (*r*(31) = 0.16, *p* = 0.37) GDTs. Furthermore, the normalized MP detection threshold did not correlate significantly with the normalized GDT in the same mode (*r*(31) = 0.16, *p* = 0.37). These correlations, as well as being nonsignificant, were also significantly smaller than those between modes for each of the two tasks: for example, gap detection in pTP mode correlated significantly less with stimulus detection in pTP mode than with gap detection in MP mode (*p* < 0.02, two-tailed). It is therefore worth noting that the lack of correlation between stimulus-detection and gap-detection tasks occurred even though our design was sensitive enough to reveal highly significant correlations between modes for each of the two tasks separately.

We also investigated whether there was a significant between-task correlation for each subject individually, by combining the relative pTP and MP thresholds in the two tasks and looking for an across-electrode correlation; the direction of this correlation differed across listeners and in no case was its significance sufficiently strong to survive the Bonferroni correction for multiple comparisons.

## **DISCUSSION**

### Signal Detection Thresholds

Signal detection thresholds are higher and more variable when obtained with the pTP compared with the MP configuration, which is consistent with previous studies (Pfingst and Xu 2004; Bierer 2007; Long et al. 2014). However, pTP thresholds do not show a significantly larger SDR than MP thresholds, because, on average, their within-channel standard deviations are numerically larger and quite variable across subjects. This could be simply due to the slope of the psychometric function being shallower in pTP mode such that a larger level change is needed for a given change in detectability. In contrast, Pfingst and Xu (2004) did show a significant difference using a related measure; they calculated the ratio of the across-electrode standard deviation in bipolar compared to MP modes and showed that this ratio was significantly greater than that for the within-electrode standard deviation. As our result was in the same direction, albeit not significant, the possibility still exists that a significant difference would be observed with more electrodes because we tested only four to six electrodes per listener.

### Gap-Detection Thresholds

Garadat and Pfingst (2011) recently studied gap-detection threshold (GDT) across electrodes in CI listeners. They reported substantial differences across electrodes when equated in percent of dynamic range, but this variation was greatly reduced when the stimulation levels for each electrode were equated for loudness. GDTs were uniformly low (<5 %) at MCL, in contrast to our finding of a very large range of GDTs in both MP and pTP modes. We observed cases in each mode where the difference across electrodes was about 25 ms within a single ear.

The present study also compared the across- and within-channel variability in the two modes for GDTs and found that neither the across- nor the within-electrode standard deviations were significantly larger for pTP than for MP and that the SDR was very similar in the two modes (Table 4).

**TABLE 4:** Across- and within-electrode standard deviations, and the ratios between them, for GDTs

	Partial tripolar (*σ* = 0.75) gap-detection threshold (ms)			Monopolar (*σ* = 0) gap-detection threshold (ms)		
Subject	Within-channel	Across-channel	Std Dev	Within-channel	Across-channel	Std Dev
	Std Dev	Std Dev	Ratio	Std Dev	Std Dev	Ratio
S22	0.55	0.55	1.00	0.49	0.24	0.49
S27	0.48	0.37	0.77	0.38	1.07	2.80
S28	4.80	9.17	1.91	4.26	11.19	2.62
S30	2.99	11.74	3.93	0.26	1.45	5.52
S39	0.23	0.20	0.85	0.21	0.22	1.07
C1	1.28	2.67	2.09	0.67	0.50	0.75
C2	1.90	4.12	2.17	1.01	3.34	3.29
C3	0.65	2.29	3.54	0.38	1.31	3.42
C4	0.87	1.05	1.20	0.82	1.08	1.33
C5	1.06	2.35	2.22	0.64	1.07	1.67
Average	1.48	3.45	1.97	0.91	2.15	2.30

### Other Suprathreshold Tasks

Pfingst (2011) has shown that modulation detection thresholds (MDTs) vary across electrodes in both bipolar (BP) and MP modes. As with the GDTs of the present study, no correlation was observed between across-site patterns of MDTs and signal detection thresholds. Note that Pfingst equated MDTs across electrodes when stimulated at the same percentage of dynamic range, rather than at equal loudness. Indeed, Garadat et al. (2012) cite a preliminary report that GDTs and MDTs correlate across electrodes. It is therefore possible that, similar to the finding of Garadat and Pfingst (2011) for GDTs, the MDTs for different electrodes would have been more similar if equated for loudness. That is, the across-electrode variation in MDTs that they report may have been mediated by loudness differences. This is unlikely to have been the case for our GDTs, where we found substantial across-electrode differences even at equal loudness (MCL). It is also worth noting that, as argued in the Introduction, MDTs measured at low modulation rates (e.g., 10 Hz; (Garadat et al. 2012)) are likely to be limited by amplitude resolution rather than by temporal resolution. Additional evidence for a qualitative difference between MDTs and GDTs is that MDTs increase with increasing pulse rate whereas the latter decrease (Kirby and Middlebrooks 2010). Note the results from the present study are also unlikely to be due to residual loudness differences at MCL, as we observed similar patterns in pTP and MP modes.

Rate discrimination has also been shown to vary significantly across electrodes for a given subject. Kong et al. (2009) measured rate discrimination for baseline rates between 100 and 500 pps and found substantial across-electrode differences in performance and in the way that it varied with baseline rate. Carlyon et al. (2010) measured rate discrimination for baseline rates of 100 and 200 pps for one basal and one apical electrode per subject and found the effect of the relative level of the signal and standard varied idiosyncratically across electrodes, subjects, and baseline rate; however, whenever there was an effect of level on performance, it was usually in the same direction for BP and MP modes. This is similar to the present findings in that performance in a suprathreshold task varied across electrodes in the same way with MP and focused stimulation. Furthermore, both tasks require good temporal resolution, and the range of rates studied by Kong et al. (2009) and by Carlyon et al. (2010) corresponded to periods between 10 and 2 ms for rates of 100 and 500 pps that fell within the range of GDTs observed here. Hence, it is possible that across-electrode variations in gap detection and rate discrimination are mediated by similar mechanisms.

### Biological Basis of Across-Electrode and Across-Mode Differences in GDTs

In principle, differences in the distance between the stimulating electrode and the target neurons could affect GDTs. Mino et al. (2004) modeled the temporal jitter in spike times for a simulated axon containing 50 nodes of Ranvier, as a function of the electrode-neuron distance. Jitter, defined as the standard deviation of spike times, increased with electrode-neuron distance because two factors increased. These were the range of nodes at which the action potential (“spike”) was initiated, and the standard deviation of spike times for a given initiation site. Jitter also decreased with increases in firing efficiency (FE; the proportion of single-pulse presentations that led to a spike). For the range of conditions that they described—electrode-neuron distances from 1 to 7 mm, and FE = 0.5 or 0.99— the jitter values were less than 0.1 ms and hence much smaller than the range of GDTs observed here. This suggests that the variation in spike times within this range, caused by variations in electrode-neuron distance, is unlikely to account for the very large GDTs observed here for some electrodes and subjects. There are at least two *caveats* to this conclusion. First, jitter increased with decreasing FE, and it is possible that the FEs for auditory nerve fibers in our (human) subjects were substantially lower than the range modeled by Mino et al. (2004). However, McKay and McDermott (1998) modeled the effect of pulse rate and level on thresholds and loudness in human CI users and concluded that FE was not very low, ranging from 0.4 to 0.9. Second, it is possible that more distant electrodes would increase jitter across the ensemble of responding neurons, due to the greater longitudinal current spread along the cochlea. Evidence against this latter conjecture comes from the fact that our GDTs were lower in MP than in pTP mode, despite the broader current spread presumably produced by MP stimulation.

An alternative explanation comes from evidence that the state of surviving neurons can affect the temporal response of auditory nerve fibers. Shepherd and Javel (1997) reported that the deafened auditory nerve could produce a “bursting” response to high-rate electrical stimulation and showed that this occurred for cats that had been deaf for 2 months, but not in a cat that was deafened immediately prior to testing. Duration of deafness also affects the refractory properties of auditory neurons, but, at least when measured using two-pulse stimuli, the increase in refractory time over a 12-month period is less than 0.5 ms (Shepherd et al. 2004). Another way in which neural survival could affect the temporal representation of pulse trains comes from its effects on the number of neurons responding to each pulse and on the level of the pulse relative to the dynamic range (DR) of each neuron. As McKay and McDermott (1998) and Botros and Psarros (2010) have pointed out, a given loudness could arise either from a small number of neurons responding high on their DRs or from a larger number of neurons responding low on their DRs. It is possible that the central auditory system improves temporal acuity by combining information from multiple neurons. This idea is consistent with the reduction in GDTs with increasing level (Garadat and Pfingst 2011). Furthermore, if the spread of excitation is broader with MP than with focused stimulation, it is also consistent with our own finding of lower GDTs with monopolar than with partial-tripolar stimulation, and with Middlebrooks’ (2008) finding that phase locking to modulation rates between 24 and 60 Hz, as measured in the auditory cortex of anesthetized guinea pigs, was more accurate for MP than for bipolar stimulation.

### Between-Subject and Between-Ear Differences in Gap Detection

**FIG. 6:**
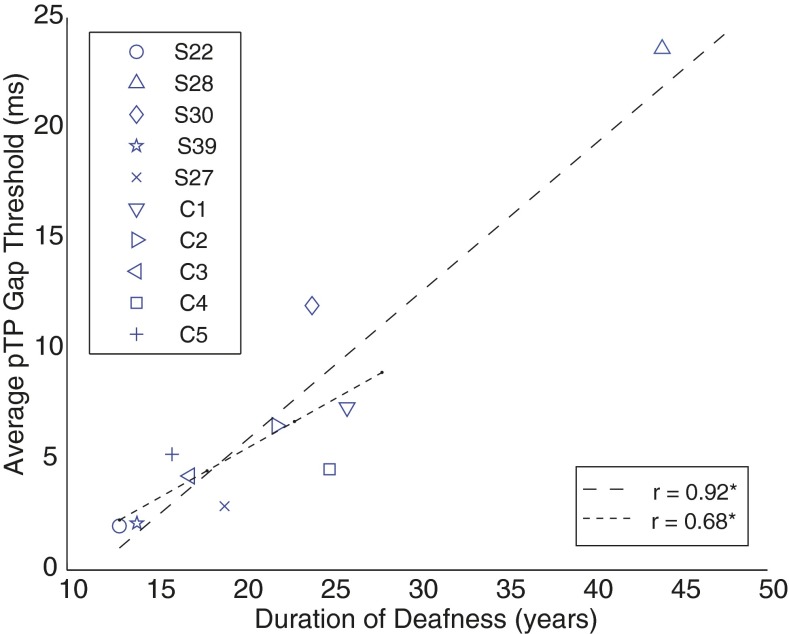
The average GDTs for each subject using the pTP configuration (*ordinate*) are plotted as a function of duration of deafness (*years*). Subject is indicated by *symbol*. The *dashed lines* are least-square fits to the data. The *longer dashes* indicate all subjects were included in the fit, and the *short dashed line* indicates that subject S28 (the outlier) and C2 (tested at soft sensation levels) were excluded from the fit and the statistics.

The present article focuses on differences in performance on psychophysical tasks across electrodes in each listener, rather than on differences between listeners. We believe this to be the more informative approach, because differences between listeners arise from a number of processes, including differences in peripheral and central auditory processes and on cognitive factors such as the ability and willingness to concentrate on repetitive tasks. Nevertheless, it is worth noting that GDTs, averaged across electrodes, did correlate with the duration of deafness, as shown in Figure 6. The correlation, which was highly significant when all subjects were included in the analysis (*r*(8) = 0.93, *p* < 0.001), also persisted when one potential outlier, subject S28, was removed (*r*(7) = 0.70, *p* < 0.05). Although we do not know the stage of auditory processing responsible for this correlation, and whether the same mechanism underlies the within- and between-subject correlations, it is worth noting that, for the bilaterally implanted listener, average GDTs were substantially larger (12.0 ms) in the ear that had been deaf for 24 years (“S30”) than in the ear that had been deaf for only 14 years (“S39,” 2.1 ms).

### Practical Implications

Our results show that GDTs could be used to observe reliable across-electrode differences, even using the MP mode and when all electrodes are stimulated at the same (comfortable) loudness. As not all clinically available devices support focused stimulation at this time, the gap detection measure could be used with the MP mode in all devices. Indeed, when the within-electrode standard deviations were taken into account, the across-electrode variation in GDTs was not significantly smaller in MP than in pTP mode. This latter observation also applied to detection thresholds, suggesting that there may not be substantial advantages to focused stimulation, at least for the tasks used here, when searching for across-electrode differences.

The results also indicate that GDTs reveal a source of across-electrode variation that is separate from that observed in signal detection thresholds. That finding is consistent with signal detection varying largely because of electrode-neuron distance and gap detection varying more from neural viability (but see Long et al. 2014). Alternatively, as noted in the Introduction, it may be that stimulation is limited by some aspect of neural survival that is different from that responsible for the variation in GDTs. In either case, gap thresholds could potentially provide a measure of neural health or viability that is different from that available from detection thresholds. Future studies using CT imaging to estimate the electrode-neuron distance may shed light on the feasibility of using GDTs in this manner. The clinical usefulness of GDTs could be assessed by close examination of confusion matrices for speech to determine if some subjects make confusions on features conveyed by electrodes that have long gap thresholds. Alternatively, one could measure speech perception for programs for which electrodes with long GDTs are deactivated and compare those to programs that include all electrodes or a different subset of electrodes and/or where electrodes with high detection thresholds are de-activated. Finally, the patterns of across-channel variability in GDTs and SDTs might be different because the two tasks are mediated by different central mechanisms.

Regardless of the diagnostic importance of GDTs or the source of variability, some of the GDTs obtained in our subjects are very long (up to 37 ms) and likely to impair transmission of important speech cues, for example, voice onset time. In everyday listening, many electrodes will convey a given distinction, but there remains the possibility that these electrodes with long GDTs will degrade listeners’ estimates of phonetic features. Note also that GDTs in this study were measured mostly at MCL and that previous studies have shown that GDTs are even longer at lower sensation levels (van Wieringen and Wouters 1999; Garadat and Pfingst 2011).

## Abbreviations

(CI): Cochlear implant
(MP): Monopolar
(pTP): Partial tripolar
(GDT): Gap-detection threshold
(T): Threshold
(MCL): Most comfortable level
(MDT): Modulation detection thresholds
(AN): Auditory nerve
(SDR): Standard deviation ratio
(ANCOVA): Analysis of Covariance
